# Development of a sensitive, specific reverse transcriptase polymerase chain reaction-based assay for epithelial tumour cells in effusions

**DOI:** 10.1038/sj.bjc.6690065

**Published:** 1999-02

**Authors:** M Sakaguchi, A K Virmani, R Ashfaq, T E Rogers, A Rathi, Y Liu, D Kodagoda, H T Cunningham, A F Gazdar

**Affiliations:** Hamon Center for Therapeutic Oncology Research, Dallas, TX, USA; Department of Pathology, University of Texas Southwestern Medical Center, Dallas, TX, USA; Veterans Administration Medical Center, Dallas, Dallas, TX, USA

**Keywords:** epithelial glycoprotein 2, cytokeratin 19, reverse transcriptase polymerase chain reaction, effusion

## Abstract

We developed a sensitive and specific method for the detection of epithelial cancer cells in effusions with a two-stage molecular-based assay which combined enrichment for cancer cells by immunomagnetic bead selection and reverse transcriptase polymerase chain reaction (RT-PCR) detection of epithelial glycoprotein 2 (EGP-2) RNA. Preliminary experiments indicated that immunobead selection was essential to avoid occasional false-positive RT-PCR results, and this method detected ten breast cancer cells electively added to 10^7^ cytologically negative effusion cells. We studied 110 cases of pleural (*n* = 68) and peritoneal (*n* = 42) effusions (30 from patients with known carcinoma and 80 from those without known carcinoma), and the results were compared with cytological findings. Of 18 effusions that were cytologically positive or suspicious for malignant cells, 17 (94%) were positive for EGP-2 RNA (the one negative sample was from a patient who recently received combination chemotherapy). Of 92 cytologically negative samples, 11 (12%) were positive for EGP-2, including six patients with a history of previous or current carcinoma. Our method appears to be highly specific and increases the sensitivity of detection of malignant cells; it may be a useful adjunct to routine cytopathological examination. *© 1999 Cancer Research Campaign*


British Journal of Cancer (1999) 79(3/4), 416–422
© 1999 Cancer Research Campaign
Article no. bjoc.1998.0065

### Development of a sensitive, specific reverse transcriptase polymerase chain reaction-based assay for epithelial tumour cells in effusions

**M Sakaguchi[1], AK Virmani[1,2], R Ashfaq[2], TE Rogers[2,3], A Rathi[1], Y Liu[1], D Kodagoda[1], HT Cunningham[1] and AF Gazdar[1,2]**

[1]Hamon Center for Therapeutic Oncology Research, [2]Department of Pathology, University of Texas Southwestern Medical Center, Dallas, TX, USA; [3]Veterans Administration Medical Center, Dallas, TX, USA

**Summary** We developed a sensitive and specific method for the detection of epithelial cancer cells in effusions with a two-stage molecular-based assay which combined enrichment for cancer cells by immunomagnetic bead selection and reverse transcriptase polymerase chain reaction (RT-PCR) detection of epithelial glycoprotein 2 (EGP-2) RNA. Preliminary experiments indicated that immunobead selection was essential to avoid occasional false-positive RT-PCR results, and this method detected ten breast cancer cells electively added to $10^7$ cytologically negative effusion cells. We studied 110 cases of pleural ($n$ = 68) and peritoneal ($n$ = 42) effusions (30 from patients with known carcinoma and 80 from those without known carcinoma), and the results were compared with cytological findings. Of 18 effusions that were cytologically positive or suspicious for malignant cells, 17 (94%) were positive for EGP-2 RNA (the one negative sample was from a patient who recently received combination chemotherapy). Of 92 cytologically negative samples, 11 (12%) were positive for EGP-2, including six patients with a history of previous or current carcinoma. Our method appears to be highly specific and increases the sensitivity of detection of malignant cells; it may be a useful adjunct to routine cytopathological examination.

**Keywords:** epithelial glycoprotein 2; cytokeratin 19; reverse transcriptase polymerase chain reaction; effusion

More than 80% of human tumours are carcinomas of epithelial origin, and direct or metastatic spread to pleural and mesothelial spaces is common. It is sometimes difficult to detect epithelial cancer cells in the resultant effusions by routine cytopathological examination because of their small numbers. Currently, the sensitivity of cytopathological examination for the detection of tumour cells is in the range of one tumour cell per $10^3$–$10^5$ of total cell number (Pelkey et al, 1996). In addition, the cytological distinction of reactive mesothelial cells from malignant cells may be difficult (Tickman et al, 1990).

Recently, polymerase chain reaction (PCR)-based methodologies have been introduced as sensitive tests for the detection of rare cancer cells in peripheral blood (Datta et al, 1994), lymph nodes (Schoenfeld et al, 1994), bone marrow aspirates (Fields et al, 1996) and other clinical specimens (Trümper et al, 1994). PCR-based methods fall into three categories: (a) methods to detect cancer-specific products or genes such as mutated K-*ras* genes, which reveal the presence of circulating pancreatic cancer cells (Nomoto et al, 1996); (b) methods to detect expression of genes specific for the tissue of origin of the tumour cell, such as prostate-specific antigen, which indicates the presence of circulating or micrometastatic prostate carcinoma cells (Katz et al, 1994); and (c) methods to detect expression of epithelial cell-specific genes such as cytokeratin 19 (K19), which is indicative of circulating or micrometastatic carcinoma cells (Datta et al, 1994; Schoenfeld et al, 1994; Fields et al, 1996; Noguchi et al, 1996*a*, 1996*b*). The last approach is not restricted to one or a few tumour types, and is useful for the detection of tumour cells from many, as well as unknown, sites of origin in fluids or tissues. Recent reports indicate that epithelial glycoprotein 2 (EGP-2) is expressed on the surface of most epithelial cells and tumours (Simon et al, 1990; de Leji et al, 1994), but not by mesothelial cells (Latza et al, 1990). Although several different nomenclatures have been used for the gene and its protein product, we use the term EGP-2 as proposed by de Leij et al (1994). Several antibodies to EGP-2 cell-surface protein have been described, but the most widely utilized is the monoclonal antibody Ber-EP4 (Latza et al, 1990). Ber-EP4 immunostaining has been used for the identification of carcinomatous effusions (Sheibani et al, 1991; de Angelis et al, 1992; Illingworth et al, 1994; Maguire et al, 1994).

Sensitive methods for the gene expression of cancer cells usually rely on reverse transcriptase PCR (RT-PCR) to detect RNA transcripts. Although many reports have documented the extreme sensitivity of RT-PCR methods for the detection of low tumour burden, false-positive results may render them as unsuitable for screening approaches. False-positive results may be due to: (a) lineage infidelity (i.e. expression of rare epithelial cell transcripts by occasional non-epithelial cells); (b) detection of genomic sequences in contaminating DNA; and (c) detection of transcripts of other genes. Whereas careful primer design and testing may overcome some of these problems, ectopic expression (lineage infidelity) remains a major source of false-positive results, especially when RNA from large numbers of test cells is utilized.

*Received 24 February 1998*
*Revised 6 May 1998*
*Accepted 13 May 1998*

*Correspondence to:* AF Gazdar, Hamon Center for Therapeutic Oncology Research, UT Southwestern Medical Center at Dallas, 5323 Harry Hines Blvd, Dallas, TX 75235-8593, USA

**Table 1** Clinical diagnoses of patient population ($n$ = 110)

| Patients with known carcinoma (cytologically positive[a]/total) | | Patients without known carcinoma (cytologically positive/total) | |
|---|---|---|---|
| Lung carcinoma | 5/10 | Chronic hepatic disease | 0/13 |
| Breast carcinoma | 3/7 | Heart failure | 0/5 |
| Ovarian carcinoma | 3/4 | Lymphoma | 0/5 |
| Colorectal carcinoma | 1/3 | Pneumonia | 0/2 |
| Prostatic carcinoma | 0/2 | Renal failure | 0/2 |
| Laryngeal carcinoma | 0/1 | Gastric ulcer | 0/2 |
| Renal cell carcinoma | 0/1 | Benign ovarian tumour | 0/2 |
| Skin carcinomas (multiple) | 0/1 | Pancreatitis | 0/1 |
| Pancreatic carcinoma | 0/1 | AIDS | 0/1 |
| | | Effusion of unknown cause | 6/47 |

[a]These figures include two cases cytologically diagnosed as ‘suspicious for malignant cells’. Of the 110 samples received for testing, 68 were pleural effusions (11 of which were cytologically positive and two ‘suspicious’), whereas 42 were ascites (five of which were cytologically positive).

A recent report demonstrated expression of seven out of eight epithelial cell and tumour-linked genes (including EGP-2) in bone marrow samples from control subjects when $10^6$ test cells were utilized (Zippelius et al, 1997). Although the mechanism of lineage infidelity is unknown, it may represent occasional loss of transcriptional control.

In an effort to design a specific and sensitive test for the detection of epithelial cancer cells in effusion fluids, we developed a two-stage RT-PCR-based assay. The first step is selective enhancement of the tumour cell population using immunomagnetic beads and a monoclonal antibody to an epithelial cell-specific surface protein. Because non-epithelial cells are absent or present in low numbers after immunobead selection, immunobead RT-PCR-based detection of epithelial cell-related transcripts renders the test highly specific. In this report, we describe the application of this test to the detection of carcinoma cells in effusion fluids.

### MATERIALS AND METHODS

Our approach to the detection of micrometastases has been published in abstract form (Sakaguchi et al, 1997).

### Patient characteristics

The clinical diagnoses of the 110 patients studied are summarized in Table 1. Sixty-eight pleural and 42 peritoneal effusions were obtained between September 1996 and April 1997 from patients attending Parkland Memorial Hospital and Veteran’s Administration Medical Center Hospital in Dallas, TX, USA. Of these, 30 were from patients with prior or current epithelial cancers, including 22 pleural effusions and eight ascites. Specimens were kept refrigerated and released for laboratory studies only when cytological examination was complete (average of 2 days after removal from the patient, range 1–7 days). Specimens were collected after obtaining informed consent. Appropriate Institutional Review Board permission was obtained for these studies.

### Cell lines

Most of the tumour and B-lymphoblastoid cell lines were initiated, maintained and characterized by us as published previously (Oie et al, 1996). Some lines were obtained from the American Type Culture Collection, Rockville, MD, USA.

### Flow cytometry

EGP-2 cell-surface protein expression was determined by disaggregating cultured cells, and incubating the resultant single-cell suspension ($10^6$ cells) with 10 μg of fluorescein isothiocyanate (FITC)-conjugated Ber-EP4 monoclonal antibody (Dako, Denmark). Non-specific binding was determined by use of an isotype control. Immunoreactivity was determined by use of a FACScan flow cytometer (Becton Dickinson Immunocytometry Systems, San Jose, CA, USA).

### Preparation of cells from effusions

Fifty millilitres of the effusion was centrifuged at 500 ***g*** for 10 min at room temperature. Pellets were washed once and resuspended in phosphate-buffered saline (PBS) containing 0.01% bovine serum albumin and a cell count was obtained. Although cytological examination indicated the persistence of tumour cells in refrigerated malignant effusions for several days, we were concerned about the possibility that RNA expression was down-regulated or absent in specimens refrigerated for lengthy periods. Therefore, for specimens reaching the laboratory more than 48 h after removal from the patient, we resuspended the cell pellet in ACL4 medium (Gazdar et al, 1986) plus 5% fetal bovine serum and incubated them in a Petri dish at 37°C in 5% carbon dioxide. After overnight incubation, the cells were harvested with a cell scraper, repelleted and handled as described above. Of the 110 clinical samples tested, 19 were cultured overnight before cell harvest.

### Selection of tumour cells by immunomagnetic beads

Cells ($10^6$–$10^7$) suspended in 4 ml of PBS were incubated with 1.25 ng of Ber-EP4 monoclonal antibody at 4°C for 1 h with gentle rotation. The cells were then washed twice with PBS and centrifuged at 400 ***g*** for 5 min and resuspended in 1.8 ml of PBS in 2 ml microcentrifugation tubes. Immunobeads (Dynabeads, Dynal, Oslo, Norway) coated with sheep anti-mouse IgG1 (Fc) were added at a cell/bead ratio of 5:1 and incubated at 4°C for 1 h with gentle rotation. Cells specifically bound to immunobeads were selected by three cycles of washing with PBS and magnetic attraction (as per manufacturer’s instructions and equipment). After initiation of these studies, immunobeads directly labelled with BER-EP4 were available commercially (Dynabeads anti-epithelial cell, Dynal). After confirming that both the indirect and

*© Cancer Research Campaign 1999* 

direct methods gave the same results with clinical specimens (data not shown), we substituted the directly labelled immunobeads for the last six samples.

### RNA extraction

Total RNA was isolated from the magnetically selected cells by using the Microscale RNA Extraction Kit (Clontech Laboratories, Palo Alto, CA, USA) following the manufacturer's instructions. After pelleting, RNA was dissolved in 20 μl of diethylpyrocarbonate-treated water and used for reverse transcription without further dilutions.

### Reverse transcription

Because the amount of RNA from small numbers of magnetically selected cells was too low to quantitate, the whole aliquot of RNA solution was used to synthesize cDNA. One microgram of 5′-pd$(T)_{12–18}$ –3′ primer (Pharmacia Biotech, Piscataway, NJ, USA) was added and the samples were incubated at 80°C for 10 min, and subsequently chilled on ice. The denatured RNA was mixed with a reaction mixture containing 500 U of Superscript II reverse transcriptase (Life Technologies, Gaithersburg, MD, USA), 10 μl of 5 × first-strand reaction buffer (Life Technologies), 5 μl of 0.1 M DTT (Life Technologies), 500 mM of dNTP mixture, 3 μl of Prime RNAAse inhibitor (5 Prime → 3 Prime, Boulder, CO, USA) in the 50 μl of final volume. The reaction was performed at 42°C for 1 h and then incubated at 95°C for 10 min to inactivate the enzyme.

**Table 2** EGP-2 cell-surface protein expression by cell lines as determined by flow cytometric analysis

| Cell line | No. positive/ no. tested (%) | Mean fluorescence channel[a] |
|---|---|---|
| B-lymphoblastoid | 0/10 (0) | (< 5) |
| Breast carcinoma | 9/10 (90) | 272 |
| Colon carcinoma | 12/13 (92) | 570 |
| Non-small-cell lung carcinoma | 6/10 (60) | 348 |
| Small-cell lung carcinoma | 8/9 (89) | 230 |
| All carcinomas | 35/42 (83) | 382 |

[a]Mean value of positive samples. A sample was considered positive if the mean fluorescence of the test sample was five or more channels greater than the isotype control.

### PCR

For EGP-2 PCR, 20 μl of reaction mixture containing 2.5 μl of 10 × PCR buffer (Life Technologies), 100 mM of dNTP mixture, 10 pmol of both sense and antisense primers, 1.5 mM of magnesium chloride and 2.5 U of *Taq* polymerase (Life Technologies) was added to 5 μl of the reverse-transcribed product. The sequences of the primers for EGP-2 were as follows: sense: 5′-GAACAATGATGGGCTTTATGA-3′; antisense: 5′-TGAGAATTCAGGTGCTTTTT-3′.

The resultant PCR product was 515 bases. Because the primer sequences were obtained from the published cDNA sequence

A B C

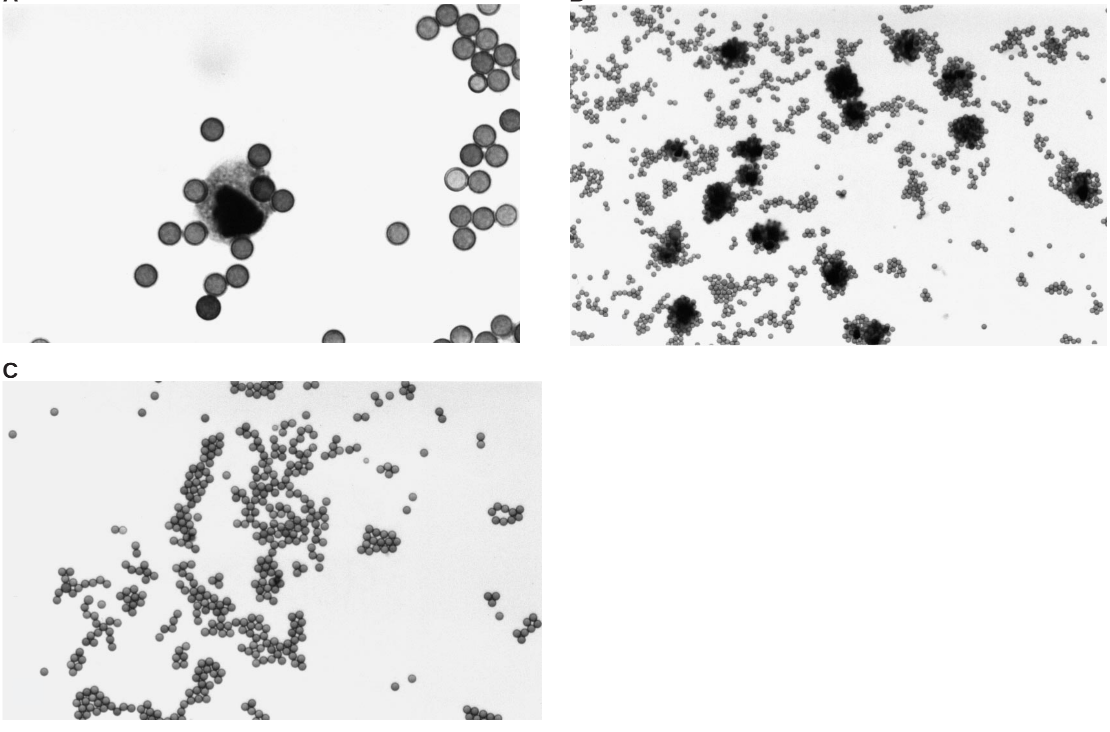

**Figure 1** Immunobead selection of carcinoma cells. Cells in a cytologically positive pleural effusion were incubated with or without Ber-EP4 followed by magnetic selection. With the use of antibody (**A** and **B**), cancer cells were selected, whereas no cells were visible when antibody was excluded (**C**). (magnification **A**, × 120; **B** and **C**, × 30)

 © Cancer Research Campaign 1999

(Simon et al, 1990), the exon(s) amplified are unknown. At the beginning of our study, we tested whether the genomic DNA could be amplified with these primers. Genomic DNA extracted from breast cancer cell lines revealed no amplification of this molecular weight. Moreover, exclusion of the reverse-transcription step resulted in failure to obtain a PCR product, thus excluding the possibility that we were amplifying genomic DNA.

After denaturation by heating the samples at 94°C for 4 min, PCR amplification was performed under the following conditions: the initial six cycles were performed with a touch-down programme at 94°C (45 s), from 67°C to 62°C with decreasing 1°C of annealing temperature per cycle (90 s), and 72°C (2 min), followed by 30 cycles of amplification at 94°C (45 s), 61°C (1 min) and 72°C (1 min) with the final extension step for 7 min at 72°C. PCR products were electrophoresed on 2% agarose gel containing ethidium bromide. The sequences of primers for K19 and the PCR conditions were as described by Noguchi et al (1996*a*).

### RESULTS

### Expression of EGP-2 RNA and protein by cultured carcinoma cells

Using a large panel ($n$ = 85) of cultured epithelial tumour cell lines, EGP-2 was expressed in 100% of all small-cell and non-small-cell lung ($n$ = 40), breast ($n$ = 20), colorectal ($n$ = 21) and miscellaneous ($n$ = 4) cancers when 10 ng of RNA was used. Ten B-lymphoblastoid lines were negative for expression when 10 ng of RNA were utilized, but positive for expression when 1 μg of RNA was used.

By flow cytometric analysis, using antibody Ber-EP4, 35 (83%) of 42 carcinoma cell lines expressed EGP-2 cell-surface protein, whereas all ten B-lymphoblastoid cell lines were negative (Table 2). We arbitrarily designated cell lines whose mean fluorescence channel was greater than 100 as high expressors, and those whose mean channel number was less than 100 as low expressors. Of the 35 positive cell lines, 28 (80%) were high expressors. High- and low-expressor breast carcinoma cell lines MCF7 (mean fluorescence channel 475) and HCC1187 (mean fluorescence channel 61) were selected for further studies (see below). Both cell lines expressed K19 and EGP-2 when concentrations of 10 and 100 pg, respectively, of total RNA were tested (data not shown).

### Immunobeads enrichment of tumour cells

To confirm that immunobead selection was enriching for tumour cells, known cytologically positive effusion samples were incubated with or without Ber-EP4 antibody followed by magnetic selection, and stained with Papanicolau. Cells with malignant cytological features were selected from the antibody-coated samples, whereas no cells were detected in the samples incubated without Ber-EP4 (Figure 1).

### Detection of K19 expression

To determine the minimum RNA concentration detected by our RT-PCR conditions, total RNA extracted from breast cancer cell line, MCF-7, was serially diluted ten fold and amplified by RT-PCR as described above. RT-PCR for K19 generated a 460-bp amplicon and the band was detected when concentrations equal or greater than 10 pg of total RNA extracted from cultured MCF-7 breast cancer cells were used. Initial RT-PCR detection experiments were performed using pelleted cells (1 × $10^7$) from seven effusions (five cytologically negative from patients not known to have cancer, and two cytologically positive samples from known cancer patients). K19 expression was present in all seven samples before selection, and in the two cytologically positive and three of the five cytologically negative samples after immunobead selection (Figure 2).

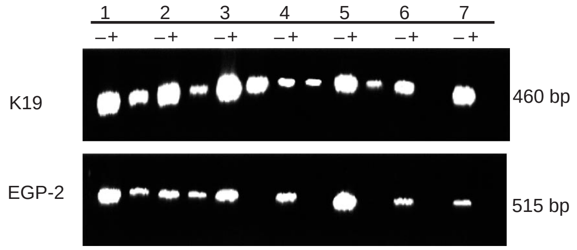

**Figure 2** Comparison of K19 and EGP-2 RT-PCR with or without immunobead selection. Both of the RT-PCR products with (+) and without (–) immunobead selection of two cytologically positive samples (sample nos. 1 and 2) and negative samples (sample nos. 3–7) were electrophoresed in parallel

**Table 3** Comparison between cytopathological examination and RT-PCR for K19 and EGP-2 for the detection of carcinoma cells in effusions

| | Cytological diagnosis ($n$) | RT-PCR results | |
|---|---|---|---|
| | | K19 positive (%) | EGP-2 positive (%) |
| Patients with known carcinoma ($n$ = 30) | Positive (11) | 11 (100) | 10 (91) |
| | Suspicious (2) | 2 (100) | 2 (100) |
| | Negative (17) | 13 (76) | 6 (35) |
| Patients without known carcinoma ($n$ = 80) | Positive (5) | 5 (100) | 5 (100) |
| | Negative (75) | 40 (53) | 5[a] (7) |
| Total subjects analysed ($n$ = 110) | Positive (16) | 16 (100) | 15 (94) |
| | Suspicious (2) | 2 (100) | 2 (100) |
| | Negative (92) | 53 (58) | 11 (12) |

[a]One subject diagnosed with metastatic carcinoma of unknown origin 1 month later.

### Detection of EGP-2 expression

RT-PCR for EGP-2 generated a 515-bp amplicon and the band was detected when concentrations equal or greater than 100 pg of total RNA extracted from MCF-7 cells were utilized for RT-PCR. Without immunobead selection, all five cytologically negative samples were positive for EGP-2 expression, although they all were negative after immunobead selection, EGP-2 expression was present in the two cytologically positive samples before and after immunobead selection (Figure 2).

To determine the sensitivity of tumour cell detection, we added various concentrations of low-EGP-2-expressing HCC1187 breast cancer cells into cytologically negative effusion samples. After immunobead selection, EGP-2 mRNA expression could be detected at a sensitivity of one tumour cell per $10^6$ effusion cells (Figure 3).

© Cancer Research Campaign 1999

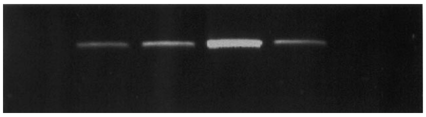

**Figure 3** EGP-2 RT-PCR after immunobead enrichment. No, 10, $10^2$ and $10^3$ breast cancer cells were spiked into $1 \times 10^7$ cells prepared from a cytologically negative pleural effusion. Positive (P) and negative (N) controls were 100 pg of RNA from MCF-7 and water blank for RT respectively

### K19 and EGP-2 expression in clinical samples

Based on the above-mentioned results, we tested 110 clinical effusion samples for K19 and EGP-2 expression after immunobead selection, and the results were compared with the cytological diagnoses (Table 3). Of these 110 samples, 68 were pleural effusions (11 of which were cytologically positive and two 'suspicious'), whereas 42 were ascites (five of which were cytologically positive). Laboratory staff were blinded as to cytological results until after completion of the assays.

There were 18 cases which were cytologically positive ($n = 16$) or suspicious ($n = 2$) for the presence of carcinoma cells, and 92 cytologically negative cases. All 18 cytologically positive or suspicious cases were positive for K19 expression, whereas 53 (58%) of the cytologically negative cases were also positive. EGP-2 expression was present in 17 (94%) of 18 cytologically positive or suspicious cases, and in 11 (12%) of cytologically negative cases. The single cytologically positive/EGP-2 expression negative sample was from a patient with breast cancer who had received multiagent chemotherapy within the previous 3 weeks and the cellularity of the effusion was low. Of the 11 cytologically negative/EGP-2-positive cases, six patients had a history of prior or present carcinoma, three of whom currently had extensive metastatic disease. The results of follow-up of the remaining five cytologically negative/EGP-2-positive cases were as follows: case 1 was diagnosed with carcinoma of unknown site 1 month later; case 2 died 1 month later with hepatic failure of unknown cause; case 3 was diagnosed with non-Hodgkin's lymphoma 14 months later; case 4 is alive without evidence of cancer 10 months later; and case 5 was lost to follow-up. Our interpretation of these five outcomes is that the results of the molecular assay were probably accurate in case 1, and could not be interpreted with certainty in the other cases.

### DISCUSSION

We developed a PCR-based assay for expression of the epithelial-associated gene products K19 and EGP-2 for the detection of epithelial cancer cells in effusions. Because preliminary experiments demonstrated that both K19 and EGP-2 were frequently expressed in cell pellets from both cytologically positive and negative specimens, we utilized a preliminary step using immunobead selection to capture carcinoma cells expressing EGP-2 cell-surface protein. Experiments with cell lines indicated that most epithelial carcinoma cell lines expressed EGP-2 cell-surface protein, whereas lymphoblastoid cell lines lacked protein expression. At high RNA concentrations (1 μg), both carcinoma and lymphoblastoid lines expressed EGP-2 RNA; however, at lower concentrations (10 ng) expression was absent in lymphoblastoid lines. More recently, without immunobead selection, in terms of EGP-2 expression, it has been also demonstrated that bone marrow aspirates (Helfrich et al, 1997) or peripheral blood cells (de Graaf et al, 1997) could yield false-positive reactions by sensitive RT-PCR methods even with samples obtained from normal volunteers. By flow cytometric examination, 83% of epithelial cell lines expressed EGP-2 cell-surface protein, usually at relatively high levels, whereas B-lymphoblastoid lines were uniformly negative. We interpret these results to indicate that lymphoblastoid cells may express EGP-2 'illegitimately' when high concentrations of RNA are used, and that some form of enrichment is required for the detection of epithelial cells when present in low concentrations in marrow, blood or effusions.

Cytokeratins are one of the most widely utilized molecular markers for the detection of epithelial cancer cells in blood, bone marrow aspirates and lymph nodes by molecular methods (Datta et al, 1994, Schoenfeld et al, 1994; Fields et al, 1996; Noguchi et al, 1996*a*, 1996*b*). In general, the three most primitive keratins, K8, K18 and K19, are expressed by all epithelial cells (Traweek et al, 1993). However, although K19 expression is the most epithelial cell-specific of the primitive keratins (Traweek et al, 1993), expression has been described in haematopoietic cells obtained from normal volunteers (Burchill et al, 1995; Krisman et al, 1995). Mesenchymally derived mesothelial cells are biphasic, exhibiting both fibroblastoid and epitheloid morphologies. They may express both keratins and vimentin (Wu et al, 1982), the intermediate filaments of epithelia and mesenchyme respectively. Our studies confirmed that K19 is expressed by the cells present in most cytologically positive as well as negative effusions. For these reasons, K19 expression is not useful for the detection of malignant epithelial cells in effusions.

As with K19, EGP-2 is widely expressed, usually in high abundance, by normal and malignant epithelial cells. However, unlike K19, EGP-2 protein is present on the cell surface and is not expressed by mesothelial cells. Thus, immunobead reaction with anti-EGP-2 antibody may be utilized for selective enrichment of carcinoma cells from malignant effusions. A similar approach has been utilized by others for the selection of circulating colorectal or breast carcinoma cells from peripheral blood (Hardingham et al, 1993, 1995, Eaton et al, 1997).

As a result of our preliminary experiments, we utilized immunoselection using magnetic beads and cells coated with Ber-EP4, a mouse monoclonal antibody against EGP-2, to selectively enrich for carcinoma cells, followed by RT-PCR-based detection of EGP-2 expression. Utilizing a low-EGP-2-expressing breast carcinoma cell line, we demonstrated that the two-step assay could detect ten tumour cells added to $10^7$ cytologically negative effusion cells. Because the selective enrichment for epithelial cells removed most or all of the mesothelial cells, 'false-positive' expression of EGP-2 by high-density mesothelial cells was reduced or eliminated. The pathology laboratories contributing specimens to this study refused to release them until they had been finally signed out, frequently resulting in a delay of 2 or more days until laboratory studies could be initiated. Although we determined that cell viability was maintained in such specimens, we were concerned that RNA levels may decrease during refrigeration. Thus, for specimens in which there was a delay longer than 48 h (19 cases), we incubated the cell pellets overnight in culture medium at 37°C before harvesting.

We tested 110 effusions, both pleural and peritoneal, of which 18 were cytologically positive or suspicious for the presence of malignant epithelial cells. EGP-2 RNA expression was detected in 17 (94%) of the positive/suspicious samples. The one 'false-negative'

© Cancer Research Campaign 1999

sample was from a patient with breast cancer who had received multiagent chemotherapy recently, and whose effusion was hypocellular. Of the 92 cytologically negative samples, EGP-2 RNA expression was detected in 11 (12%). Of these, six were from patients with known previous or present carcinoma, whereas the other five were from patients without known or suspected carcinoma at the time of analysis. One subject was diagnosed with metastatic carcinoma of unknown origin 1 month later. Details of the outcomes of the others and our interpretations of these findings are presented in the Results section.

The true sensitivity and specificity of our assay cannot be ascertained with complete accuracy when compared with cytological examination. If we presume that the two ‘cytologically suspicious’ samples were really positives, the sensitivity of the molecular assay was 17 out of 18 (94%). The true specificity of our assay is even more difficult to determine. If we presume that all of the cytologically negative specimens from cancer patients contained malignant cells (including the patient who was diagnosed a month later), as did the two cytologically ‘suspicious’ samples, the specificity of the assay would be 19 out of 23 (83%) or better.

To the best of our knowledge, no molecular-based assay has been utilized for the detection of malignant effusions. The molecular examination we have developed appears to be a useful adjunct to routine cytological examination of carcinomatous effusions, especially in cases having low numbers of tumour cells or in cases in which the cytological interpretation is difficult. Our findings will have to be confirmed and extended in a prospective study specifically targeting a tumour patient population.

### ACKNOWLEDGEMENTS

We thank Dr AN Makarovskiy, Department of Urology, Rhode Island Hospital, Providence, Rhode Island, USA, for advice on methodology, and Dr Eugene Frenkel for encouragement and specimens. Dr David Euhus assisted with collection of patient data. This study was supported by grant ATP-003660-073 from the Advanced Technology Program, the Texas Higher Education Coordinating Board, State of Texas and the Specialized Program of Research Excellence (SPORE) grant (1-P50-CA70907-01) from the National Cancer Institute, Bethesda, MD, USA.

### REFERENCES

Burchill SA, Bradbury MF, Pittman K, Southgate J, Smith B and Selby P (1995) Detection of epithelial cancer cells in peripheral blood by reverse transcriptase-polymerase chain reaction. *Br J Cancer* **71**: 278–281

Datta YH, Adams PT, Drobyski WR, Ethier SP, Teny VH and Roth MS (1994) Sensitive detection of occult breast cancer by the reverse-transcriptase polymerase chain reaction. *J Clin Oncol* **12**: 475–482

de Angelis M, Buley ID, Heryet A and Gray W (1992) Immunocytochemical staining of serous effusions with the monoclonal antibody Ber-EP4. *Cytopathology* **3**: 111–117

de Graaf H, Mælandsmo GM, Ruud P, Forus A, Øyjord T, Fodstad Ø and Hovig E (1997) Ectopic expression of target genes may represent an inherent limitation of RT-PCR assays used for micrometastasis detection: studies on the epithelial glycoprotein gene *EGP-2*. *Int J Cancer* **72**: 191–196

de Leij L, Helrich W, Stein R and Mattes MJ (1994) SCLC-cluster-2 antibodies detect the pancarcinoma/epithelial glycoprotein EGP-2. *Int J Cancer* (suppl.) **8**: 60–66

Eaton MC, Hardingham JE, Kotasek D and Dobrovic A (1997) Immunobead RT-PCR: a sensitive method for detection of circulating tumor cells. *Biotechniques* **22**: 100–105

Fields KK, Elfenbein GJ, Trudeau WL, Perkins JB, Janssen WE and Moscinski LC (1996) Clinical significance of bone marrow metastases as detected using the polymerase chain reaction in patients with breast cancer undergoing high-dose chemotherapy and autologous bone marrow transplantation. *J Clin Oncol* **14**: 1868–1876

Gazdar AF and Oie HK (1986) Growth of cell lines and clinical specimens of human non-small cell lung cancer in a serum-free defined medium (letter to the editor). *Cancer Res* **46**: 6011

Hardingham JE, Kotasek D, Farmer B, Butler RN, Mi J-X, Sage RE and Debrovic A (1993) Immunobead-PCR: a technique for the detection of circulating tumor cells using immunomagnetic beads and the polymerase chain reaction. *Cancer Res* **53**: 3455–3458

Hardingham JE, Kotasek D, Sage RE, Eaton MC, Pascoe VH and Dobrovic A (1995) Detection of circulating tumor cells in colorectal cancer by immunobead-PCR is a sensitive prognostic marker for relapse of disease. *Mol Med* **1**: 789–794

Helfrich W, ten Poele R, Meersma GJ, Mulder NH, de Vries EGE, de Leij L and Smit EF (1997) A quantitative reverse transcriptase polymerase chain reaction-based assay to detect carcinoma cells in peripheral blood. *Br J Cancer* **76**: 29–35

Illingworth AL, Young JA and Johnson GD (1994) Immunofluorescent staining of metastatic carcinoma cells in serous fluid with carcinoembryonic antibody, epithelial membrane antibody, AUA-1 and Ber-EP4. *Cytopathology* **5**: 270–281

Katz AE, Olsson CA, Raffo AJ, Cama C, Perlman H, Seaman E, O’toole KM, McMahon D, Benson MC and Buttyan R (1994) Molecular staging of prostate cancer with the use of an enhanced reverse transcriptase-PCR assay. *Urology* **43**: 765–775

Krismann M, Todt B, Schröder J, Gareis D, Müller K-M, Seeber S and Schütte J (1995) Low specificity of cytokeratin 19 reverse transcriptase-polymerase chain reaction analyses for detection of hematogenous lung cancer dissemination. *J Clin Oncol* **13**: 2769–2775

Latza U, Niedobitek G, Schwarting R, Nekarda H and Stein H (1990) Ber-EP4: new monoclonal antibody which distinguishes epithelia from mesothelial. *J Clin Pathol* **43**: 213–219

Maguire B, Whitaker D, Carrello S and Spagnolo D (1994) Monoclonal antibody Ber-EP4: its use in the differential diagnosis of malignant mesothelioma and carcinoma in cell blocks of malignant effusions and FNA specimens. *Diagn Cytopathol* **10**: 130–134

Noguchi S, Aihara T, Motomura K, Inaji H, Imaoka S and Koyama H (1996*a*) Histologic characteristics of breast cancers with occult lymph node metastases detected by Keratin 19 mRNA reverse transcriptase-polymerase chain reaction. *Cancer* **78**: 1235–1240

Noguchi S, Hiratsuka M, Furukawa H, Aihara T, Kasugai T, Tamura S, Imaoka S, Koyama H and Iwanaga T (1996*b*) Detection of gastric cancer micrometastasis in lymph nodes by amplification of keratin 19 mRNA with reverse transcriptase-polymerase chain reaction. *Jpn J Cancer Res* **87**: 650–654

Nomoto S, Nakao A, Kasai Y, Harada A, Nonami T and Takagi H (1996) Detection of ras gene mutations in perioperative peripheral blood with pancreatic adenocarcinoma. *Jpn J Cancer Res* **87**: 793–797

Oie HK, Russell EK, Camey DN and Gazdar AF (1996) Cell culture methods for the establishment of the NCI series of lung cancer cell lines. *J Cell Biochem* (suppl.) **24**: 24–31

Pelky TJ, Frierson HF and Bruns DE (1996) Molecular and immunological detection of circulating tumor cells and micrometastases from solid tumors. *Clin Chem* **42**: 1369–1381

Sakaguchi M, Virmani A, Yashima K, Makarovskiy AN, Ashfaq R and Gazdar A (1997) Development of a sensitive, specific RT-PCR based assay for the detection of epithelial cells and its application to effusions. *Proc Am Assoc Cancer Res* **38**: 570

Schoenfeld A, Luqmani Y, Smith D, O’Reilly S, Shousha S, Sinnet HD and Coombes RC (1994) Detection of breast cancer micrometastases in axillary lymph nodes by using polymerase chain reaction. *Cancer Res* **54**: 2986–2990

Sheibani K, Shin SS, Kezirian J and Weiss LM (1991) Ber-EP4 antibody as a discriminant in the differential diagnosis of malignant mesothelioma versus adenocarcinoma. *Am J Surg Pathol* **15**: 779–784

Simon B, Podolsky DK, Moldenhauer G, Isselbacher KJ, Gattoni-Celli S and Brand SJ (1990) Epithelial glycoprotein is a member of a family of epithelial cell surface antigens homologous to nidogen, a matrix adhesion protein. *Proc Natl Acad Sci USA* **87**: 2755–2759

Tickman RJ, Cohen C and Fekete PS (1990) Distinction between carcinoma cells and mesothelial cells in serious effusions. Usefulness of immunohistochemistry. *Acta Cytol* **34**: 491–496

Traweek ST, Liu J and Battifora H (1993) Keratin gene expression in non-epithelial tissues: detection with polymerase chain reaction. *Am J Pathol* **142**: 1111–1118

Trümper LH, Bürger B, von Bonin F, Hintze A, von Blohn G, Pfreundschuh M and Daus H (1994) Diagnosis of pancreatic adenocarcinoma by polymerase chain reaction from pancreatic secretions. *Br J Cancer* **70**: 278–28

© Cancer Research Campaign 1999 

Wu YJ, Parker LM, Binder NE, Beckett MA, Sinard JH, Griffiths CT and Rheinwald JG (1982) The mesothelial keratins: a new family of cytoskeletal proteins identified in cultured mesothelial cells and nonkeratinizing epithelia. *Cell* **31**: 693–703

Zippelius A, Kufer P, Honold G, Köllermann MW, Obemeder R, Schlimok G, Riethmüller G and Pantel K (1997) Limitations of reverse-transcriptase polymerase chain reaction analyses for detection of micrometastatic epithelial cancer cells in bone marrow. *J Clin Oncol* **15**: 2701–2708

*© Cancer Research Campaign 1999*
